# Comparative Study of Triboelectric Nanogenerators with Differently Woven Cotton Textiles for Wearable Electronics

**DOI:** 10.3390/polym11091443

**Published:** 2019-09-03

**Authors:** Jaebum Jeong, Jin-Hyuk Kwon, Kyungmin Lim, Swarup Biswas, Alexandra Tibaldi, Suwoong Lee, Hyun Ju Oh, Jong-Hyoung Kim, Jaehoon Ko, Dong-Wook Lee, Hanchul Cho, Philippe Lang, Jaewon Jang, Sohee Lee, Jin-Hyuk Bae, Hyeok Kim

**Affiliations:** 1 Korea Institute of Industrial Technology, 89, Yangdaegiro-gil, Ipjang-myeon, Seobuk-gu, Cheonan-si, Chungcheongnam-do 31056, Korea; 2 School of Electronics Engineering, Kyungpook National University, Daegu 41566, Korea; 3 Department of Electrical Engineering, RIGET, Gyeongsang National University, Jinju 52828, Korea; 4 ITODYS, Université de Paris 7, CNRS UMR 7086, 13 rue Jean-Antoine de Baïf, 75013 Paris, France; 5 Department of Clothing and Textiles, Research Institute of Natural Science, Gyeongsang National University, Jinju 52828, Korea; 6 Department of Electrical and Computer Engineering, University of Seoul, 163 Seoulsiripdaero, Dongdaemun-gu, Seoul 02504, Korea

**Keywords:** triboelectric nanogenerator, cotton fiber surface, woven cotton textile, output voltage

## Abstract

A comparative study of the electrical performance of triboelectric nanogenerators (TENGs) with plain- and 2/1 twill-woven cotton textiles was conducted. Furthermore, the microstructures of the cotton fiber surfaces were examined to understand the fundamental mechanical interaction among the cotton fibers in the TENGs. The TENG with 2/1 twill-woven cotton textiles exhibited higher output voltages compared to that with plain-woven cotton textiles. The difference in the output voltage between the two types of TENGs resulted from the difference in triboelectric charge generation between the constituent cotton textiles. The higher output voltage of the TENG with 2/1 twill-woven cotton textiles was attributed to the higher density in triboelectric interactions among the cotton fiber molecules.

## 1. Introduction

Our daily life is filled with diverse forms of ambient energies that are continuously generated and observable, but mostly wasted without being exploited. Various types of energy harvesters have been developed in order to harness such energies. For example, many promising technologies have been developed to convert solar and wind energies to electrical energy [1]. Following the demonstration of triboelectric nanogenerators (TENGs) consisting of polyester and Kapton polymer thin films by Wang et al. in 2012, TENGs have attracted significant interest as a promising energy harvesting technology [2,3,4,5,6]. TENGs can convert mechanical energy into electrical energy through the phenomenon termed the triboelectric effect [7,8,9]. For example, electrostatic charges are generated in our shirts, coats, and dresses by the triboelectric effect when their textiles are rubbed against each other. Interestingly, the electrostatic charges generated can be utilized as a useful energy source with the aid of TENGs. Since cotton fibers are one of the most common materials used for manufacturing clothing, it would be useful to investigate the electrical performance of cotton-textile-based TENGs by employing various approaches. For example, investigations of TENGs with differently-woven textiles can provide useful information about the suitability of different textile morphologies for energy harvesting. In the field of textile engineering, previous studies have reported that mechanical properties such as stiffness, compressibility, and tensile strength, which are associated with yarn interlacement, differ among differently-woven textiles [10,11,12]. Many different patterns, such as plain, matt, twill, warp rib, herringbone, and satin are used for weaving yarns into textiles [11]. The mechanical differences among differently-woven cotton textiles may be reflected in electrical differences among cotton-textile-based TENGs employing such textiles, since triboelectric charges can be generated by mechanical interactions such as the friction among cotton fibers. In a previous study, silver- and polytetrafluoroethylene-textile-based TENGs with different weave patterns, showing the output voltage of 23.50 V, were investigated in terms of the textiles′ mechanical properties [13]. In particular, the relationship between the mechanical properties of the cotton textiles employed and the device performance in cotton-textile-based TENGs has not been fully explored in the context of electronic engineering, despite the physical processes involved with triboelectric charge generation on organic surfaces and molecules being moderately well understood. In addition, it is noteworthy that owing to their mechanical flexibility, fiber-type TENGs are more suitable candidates for wearable electronics compared to film-type TENGs [14,15].

In this work, a comparative study of the output voltages of TENGs fabricated with plain- and 2/1 twill-woven cotton textiles was conducted. The microstructures of the cotton fibers were examined using atomic force microscopy (AFM) and scanning electron microscopy (SEM) analyses. The difference in the output voltages of the TENGs was elucidated on the basis of triboelectric charge generation in the cotton textiles.

## 2. Materials and Methods

**Figure 1:**
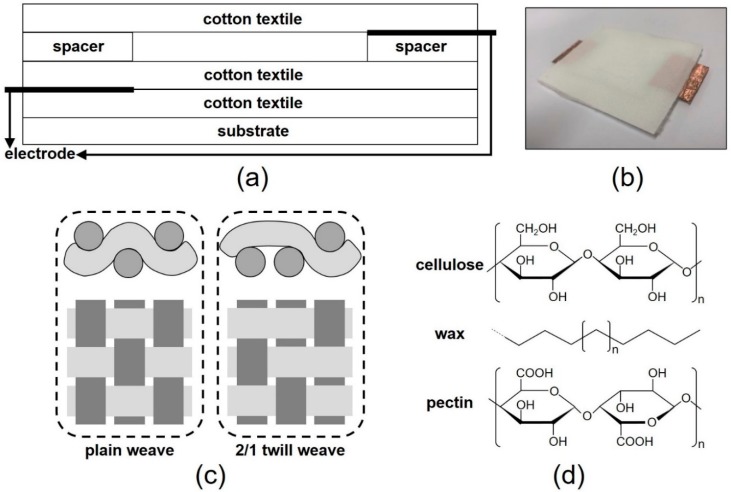
(**a**) Schematics of the cotton-textile-based triboelectric nanogenerator, (**b**) a photo of the fabricated TENG, (**c**) plain and 2/1 twill weave patterns, and (**d**) the molecular structures of cellulose, wax, and pectin.

TENGs were fabricated by using cotton textiles as active triboelectric layers. Figure 1a shows a schematic of the TENGs. For use as substrates and spacers, polydimethylsiloxane (PDMS) films were prepared using Sylgard-184, an elastomeric PDMS kit manufactured by Dow Corning (Midland, MI, USA). A 10:1 PDMS base/curing agent mixture, which was stored in a vacuum desiccator to remove air bubbles, was poured onto a flat plate and subjected to thermal treatment at 100 °C for 1 h on a hot plate. As shown in Figure 1a, cotton textiles, a PDMS spacer film, and copper-tape electrodes were stacked and fixed on a PDMS substrate using an adhesive for fabricating the TENG. A photo of the fabricated TENG is shown in Figure 1b. Plain- and 2/1 twill-woven cotton textiles were used as the active triboelectric layers. Figure 1c shows schematics of plain- and 2/1 twill-woven cotton textiles [16]. The cotton textiles consisted of warp and weft yarns, each of which was a strand of cotton fibers. The thicknesses of the PDMS spacer film, cotton textile, and copper-tape electrode were 1 mm, 280 μm and 50 μm, respectively. The PDMS spacer film had a 2.5 cm × 2.5 cm square gap. The cotton textiles were purchased from Sombe (Congo). The output voltages of the TENGs were measured using a low-noise current preamplifier (SR570; Stanford Research Systems, Sunnyvale, CA, USA).

## 3. Results and Discussion

The chemical composition of cotton fibers should be considered in order to understand triboelectric charge generation in cotton textiles. Cotton fibers contain various organic compounds, including cellulose, waxes, pectins, organic acids, and some inorganic substances; cellulose accounts for approximately 90% of the dry weight of cotton fibers [17]. Figure 1d shows the molecular structures of cellulose, wax, and pectin [18]. When mechanical stresses are applied to cotton textiles, energetic interactions may occur among the various molecules on their surface. Collisions among the different molecules are likely to induce electron exchange due to the difference in electron affinity among them [19].

**Figure 2:**
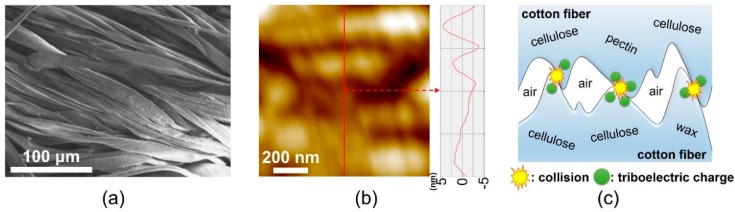
(**a**) Scanning electron microscopy image of cotton fibers, (**b**) atomic force microscopy image of a cotton fiber, and (**c**) a schematic of collisions among the molecules in the surface microstructures of the cotton fibers.

The surface microstructures of cotton fibers were examined to understand the basic mechanical interaction among the cotton fiber yarns in the TENGs. Unlike film-type TENGs based on film-to-film interactions, examining the morphological properties of cotton fibers would be important and helpful in understanding the working mechanisms of fiber-type TENGs based on fiber-to-fiber interactions. Figure 2a shows a SEM image of cotton fibers in a single yarn. The observed cotton fibers have elongated and curved shapes, which is in conformance with the general morphologies of cotton fibers [20]. Figure 2b shows an AFM image of a cotton fiber. The cotton fiber exhibited an uneven and bumpy surface; the root-mean-square surface roughness of the cotton fiber was 3.247 nm. Figure 2c shows the triboelectric interaction between the surfaces of adjacent cotton fibers. When friction occurs between adjacent cotton fibers, the molecules in the surface microstructures of the cotton fibers collide with each other, thereby generating triboelectric charges. It should be noted that air gaps are present between adjacent cotton fibers in a single yarn and between warp and weft yarns in a single textile since the fibers as well as the warp and weft yarns are not completely interfaced with each other. Presumably, triboelectric charges are generated not only at the colliding surfaces between the upper and lower textiles, but also at the colliding surfaces of cotton fibers in each yarn.

**Figure 3:**
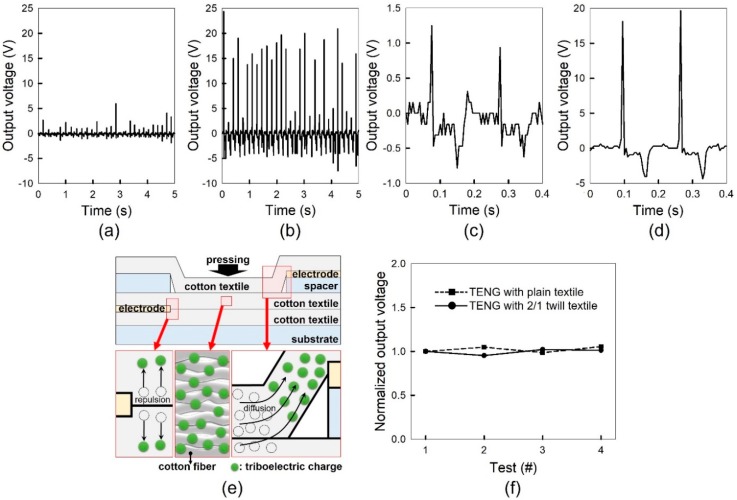
Output voltages of the triboelectric nanogenerators with (**a**) plain- and (**b**) 2/1 twill-woven cotton textiles. Two successive output voltage waveforms of the triboelectric nanogenerators with (**c**) plain- and (**d**) 2/1 twill-woven cotton textiles. (**e**) A schematic of the working mechanism of the triboelectric nanogenerator. (**f**) The normalized positive peak output voltages of Test #1, #2, #3, and #4.

The output voltages of the TENGs with plain- and 2/1 twill-woven cotton textiles were measured by applying a pressure of 0.47 N/cm^2^ on the textiles at a frequency of 5.8 Hz for 5 s. Figure 3a,b show the output voltages of the TENGs, respectively. The positive peak output voltages of the TENGs with plain- and 2/1 twill-woven cotton textiles were 1.59 ± 0.08 and 12.47 ± 0.62 V, respectively, and the negative peak output voltages were −0.86 ± 0.05 and −4.13 ± 0.21 V, respectively. These values were obtained from tens of devices for each case. Clearly, the TENG with 2/1 twill-woven cotton textiles exhibited higher peak output voltages. Figure 3c and d show the successive two output voltage waveforms of the TENGs with plain- and 2/1 twill-woven cotton textiles, respectively. Considering the stabilizing time of the measured voltage from the negative peak to 0 V, the time interval, corresponding to the 5.8 Hz measurement frequency, was properly chosen to carry out the repetitive measurement with the prevention of the electrical interference between successive measurements. A single output voltage waveform shows a positive peak resulting from pressure application and a negative peak generated by pressure release. When pressure is applied on the textiles, triboelectric charges are generated in them, as shown in Figure 3e. The generated mobile triboelectric charges, which are electrons, move along the surface of cotton fibers and are subsequently gathered near the top electrode by diffusion, resulting in a positive peak in the output voltage. Cellulose molecules would form the triboelectric charge pathway, considering that cellulose is the major component of cotton fibers and that cellulose-to-cellulose charge transfer is the electron movement between the identical energy levels of cellulose molecules. Previously, it was found that triboelectric charges can diffuse laterally on the surface of silicon dioxide which is one of the most well-known insulators with high resistivity [21]. At the tip of the bottom electrode, electric repulsion among triboelectric charges possibly hindered their accumulation near the electrode, as shown in Figure 3e. The recombination of triboelectric charges and the continuous charge flow caused the electrodes to have opposite polarities, which lead to the negative peak in the output voltage [2]. In addition, the output voltages of the TENGs were measured repeatedly with repeated press and release operations to determine the stability of voltage generation. Figure 3f shows the normalized positive peak output voltages of Test #1, #2, #3, and #4. Each test, consisting of twenty waveforms, was carried out after one thousand press and release operations. During the whole measurement, the TENGs exhibited no significant variation in the voltage generation. In other words, the TENGs generated voltage at a stable level with thousands of pressing and releasing applications. The stable voltage generation can be attributed to the chemical and morphological stabilities of cotton fibers under mechanical stresses.

**Figure 4:**
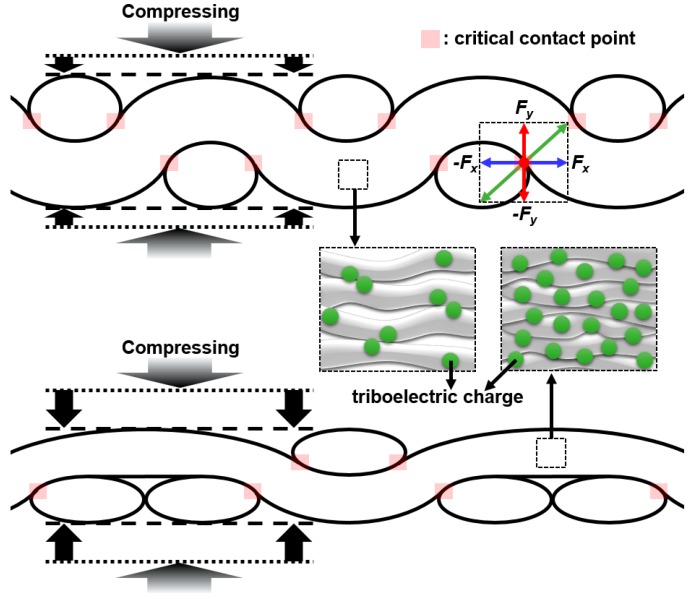
Schematics of plain- and 2/1 twill-woven cotton textiles to which compressive forces were applied.

To explain the difference in output voltage between the TENGs, an understanding of the relationship between the mechanical properties of the cotton textiles and the triboelectric charge density in the cotton textiles is necessary. Figure 4 shows schematics of plain- and 2/1 twill-woven cotton textiles to which compressive forces were applied. The textiles contain the critical contact points of warp and weft yarns, which are resistant to compressive deformation due to the vertical components of the contact forces. The difference in the density of the critical contact points would create the difference in compressibility between the two types of textiles. Note that in previous studies, the difference in compressibility between plain and twill weaves was proven experimentally [11,12]. The degree of compressive deformation in the 2/1 twill-woven cotton textiles is likely to be higher than that in plain-woven cotton textiles, leading to greater mechanical interaction among cotton fibers. Consequently, under a compressive force, the density of the generated triboelectric charge in the 2/1 twill-woven cotton textiles would be higher than that in the plain-woven cotton textiles. Thus, the different output voltages of the TENGs with plain- and 2/1 twill-woven cotton textiles appear to result from the difference in the mechanical property of the textiles. Meanwhile, immobile positive triboelectric charges presumably interfere with the flow of triboelectric electrons, restricting the performance of the TENGs. Our future work will be specific studies on the structural modifications of the TENGs such as use of mesh-type electrodes for improving electron collection efficiency.

## 4. Conclusions

TENGs were fabricated using plain- and 2/1 twill-woven cotton textiles, and their output voltages were compared. The TENGs with plain- and 2/1 twill-woven cotton textiles exhibited average positive peak output voltages of 1.59 and 12.47 V, respectively. Their average negative peak output voltages were −0.86 and −4.13 V, respectively. The difference in the output voltages is explained on the basis of the difference in the mechanical properties of plain- and 2/1 twill-woven cotton textiles. The results of this study are expected to improve our understanding of triboelectric charge generation on cotton fiber surfaces and help in designing the structure of cotton textiles from the viewpoints of triboelectric engineering and wearable electronics.

## References

[1] Vincent P., Song D.-S., Kwon H.B., Kim D.-K., Jung J.-H., Kwon J.-H., Choe E., Kim Y.-R., Kim H., Bae J.-H. (2018). Towards maximizing the haze effect of electrodes for high efficiency hybrid tandem solar cell. Appl. Surf. Sci..

[2] Fan F.-R., Tian Z.-Q., Wang Z.L. (2012). Flexible triboelectric generator!. Nano Energy.

[3] Guo H., Chen J., Tian L., Leng Q., Xi Y., Hu C. (2014). Airflow-induced triboelectric nanogenerator as a self-powered sensor for detecting humidity and airflow rate. ACS Appl. Mater. Interfaces.

[4] Seol M.-L., Woo J.-H., Jeon S.B., Kim D., Park S.J., Hur J., Choi Y.-K. (2015). Vertically stacked thin triboelectric nanogenerator for wind energy harvesting. Nano Energy.

[5] Seol M.-L., Han J.-W., Moon D.-I., Yoon K.J., Hwang C.S., Meyyappan M. (2018). All-printed triboelectric nanogenerator. Nano Energy.

[6] Mallineni S.S.K., Dong Y., Behlow H., Rao A.M., Podila R. (2017). A wireless triboelectric nanogenerator. Adv. Energy Mater..

[7] Wang Z.L., Chen J., Lin L. (2015). Progress in triboelectric nanogenerators as a new energy technology and self-powered sensors. Energy Environ. Sci..

[8] Zhao K., Wang Z.L., Yang Y. (2016). Self-powered wireless smart sensor node enabled by an ultrastable, highly efficient, and superhydrophobic-surface-based triboelectric nanogenerator. ACS Nano.

[9] Kim S., Gupta M.K., Lee K.Y., Sohn A., Kim T.Y., Shin K.-S., Kim D., Kim S.K., Lee K.H., Shin H.-J. (2014). Transparent flexible graphene triboelectric nanogenerators. Adv. Mater..

[10] Jahan I. (2017). Effect of fabric structure on the mechanical properties of woven fabrics. Adv. Res. Text. Eng..

[11] Dhoot N.S., Patil L.G., Katkar P.M. (2014). Effect of fabric weaves on compressional behaviour of woven fabric. Indian J. Fibre Text..

[12] Yousaf Z., Potluri P., Withers P.J. (2017). Influence of tow architecture on compaction and nesting in textile preforms. Appl. Compos. Mater..

[13] Kwak S.S., Kim H., Seung W., Kim J., Hinchet R., Kim S.-W. (2017). Fully stretchable textile triboelectric nanogenerator with knitted fabric structures. ACS Nano.

[14] Kwak S.S., Yoon H.-J., Kim S.-W. (2019). Textile-based triboelectric nanogenerators for self-powered wearable electronics. Adv. Funct. Mater..

[15] Kim H., Lee S. (2018). Characterization of carbon nanofber (CNF)/polymer composite coated on cotton fabrics prepared with various circuit patterns. Fash. Text..

[16] Sabuncu M., Özdemir H. (2018). Recognition of weave patterns of striped fabrics using optical coherence tomography. Fibres Text. East. Eur..

[17] Grishanov S., Clark M. (2011). Cellulosic fibres. Handbook of Textile and Industrial Dyeing.

[18] Dehghani S., Hosseini S.V., Regenstein J.M. (2018). Edible films and coatings in seafood preservation: A review. Food Chem..

[19] Lee S.-M., Sung W.-Y., Kim W.-J., Ok J.-G., Kim Y.-H. (2008). Fabrication of field emitters with carbon nanotubes using triboelectricity. Jpn. J. Appl. Phys..

[20] Kafle K., Greeson K., Lee C., Kim S.H. (2014). Cellulose polymorphs and physical properties of cotton fabrics processed with commercial textile mills for mercerization and liquid ammonia treatments. Text. Res. J..

[21] Zhou Y.S., Liu Y., Zhu G., Lin Z.-H., Pan C., Jing Q., Wang Z.L. (2013). In situ quantitative study of nanoscale triboelectrification and patterning. Nano Lett..

